# Textile Slotted Waveguide Antennas for Body-Centric Applications

**DOI:** 10.3390/s22031046

**Published:** 2022-01-28

**Authors:** Davorin Mikulić, Evita Šopp, Davor Bonefačić, Zvonimir Šipuš

**Affiliations:** Faculty of Electrical Engineering and Computing, University of Zagreb, Unska 3, 10000 Zagreb, Croatia; davorin.mikulic@fer.hr (D.M.); evita.sopp@fer.hr (E.Š.); davor.bonefacic@fer.hr (D.B.)

**Keywords:** wearable antennas, textile antennas, slotted waveguide antennas, conductive textile

## Abstract

One of the major challenges in the development of wearable antennas is to design an antenna that can at the same time satisfy technical requirements, be aesthetically acceptable, and be suitable for wearable applications. In this paper, a novel wearable antenna is proposed—textile realization of a slotted waveguide antenna. The antenna is realized using conductive fabric to manufacture the walls of a rectangular waveguide in which the slots were cut out. All connections and cuts are sewn with conductive thread taking over advantages of the traditional process of manufacturing textile objects. The developed slotted waveguide array prototype, containing three slots and designed for operation in the 5.8-GHz ISM band, is experimentally characterized and compared to an equivalent metallic antenna. The achieved operating bandwidth is larger than 300 MHz in both cases. The measured gain of a textile slotted waveguide array is around 9 dBi with a radiation efficiency larger than 50% in the whole operating bandwidth, i.e., the textile array showed a 2 dB lower gain in comparison to the metallic counterpart. The gain is stable in the whole bandwidth and the radiation patterns do not differ. The results demonstrated that such textile antennas are suitable for body-centric communication and sensor systems and can be integrated into clothing, e.g., into a smart safety vest or into a uniform. Further analysis of various realizations of slotted waveguide antennas is presented showing that different versions of the proposed antenna can be used in all three off-body, on-body, and in-body communication scenarios.

## 1. Introduction

Wireless connectivity is becoming ever more important in present-day life. The advent of the Internet-of-Things [1] and enabling technologies introduced in 5G [2] and expanded in the forthcoming 6G mobile communications foster this connectivity [3,4]. One important environment for establishing wireless connectivity are body-centric communications [1,5]. They are divided in three areas: Off-body, on-body, and in-body; each of them with its own challenges for the designers of antennas and communication systems. Body-centric communications, as any wireless system, require antennas. Here textile antennas find their application as they can be seamlessly integrated in clothing, offering the wearer freedom in daily activities while enabling the connectivity to off-body wireless access points, establishing Wireless Body Area Networks (WBAN), and communication with wearable and implantable devices and sensors. Application of such communications are numerous [5]; from rescue services, security, surveillance, and military applications, to healthcare and telemedicine, sports and entertainment.

To fulfill their task, textile antennas should resemble common textile materials in flexibility, lightweight, and aesthetics yet show good radiation efficiency and impedance matching, mechanical durability, water resistance, etc., as well as allow implementation of technologies such as Multiple-Input Multiple-Output (MIMO) [5,6,7,8,9,10,11]. As textile antennas are designed to operate on the human (user’s) body, another important requirement are maximum exposure limits to electromagnetic (EM) fields, i.e., maximum Specific Absorption Rate (SAR), which should not be exceeded [12,13,14].

While the vast majority of existing textile antennas are dipoles, monopoles, Planar Inverted-Fs (PIFAs), microstrip patches, and planar antennas in general, which are of a low profile and can be used on a variety of curved surfaces [7,8,15], there are still unexplored possibilities of adapting other types of common antennas to be realized as textile ones and adapted for wearable application. The aim of this paper is to explore properties of a textile slotted waveguide antenna array [16] that could easily be attached to a jacket, vest, belt, or another article of clothing.

A waveguide slot antenna realized of textile-filled waveguide was introduced in [17], yet the waveguide walls were realized of copper foil which is impractical for wearable applications. Some authors proposed the application of a Surface Integrated Waveguide (SIW) realized in textile as Textile Integrated Waveguides (TIW) [18,19,20,21]. However, TIW is usable at higher frequencies determined by the thickness of the used textile, while for lower frequencies multiple textile layers can result in a bulky design. Additionally, lateral walls were realized either using eyelet vias or an Electromagnetic Band-gap (EBG) structure, which can be unpractical. On the other hand, the application of a textile rectangular waveguide allows the operation of the proposed antenna in ISM and 5G frequency bands below 6 GHz without excessive increase in dimensions or weight. Furthermore, hollow waveguides have the lowest possible losses in comparison to any other transmission line realization. When the waveguide needs to be filled, the EM parameters (permittivity, losses, etc.) of the waveguide filling should be as similar to air as possible to maintain this advantage. As will be discussed later in the paper, this is possible for off-body and on-body applications, where foam-like filling would be the best. For in-body applications, the situation is completely different, and other types of filling, with EM parameters closer to the values for human tissue are needed.

In contrast to designs in [18,19,20], the considered waveguide antenna is fully realized using conductive fabric and sewing manufacturing procedure which represent the most natural way of making textile objects. In this way, the proposed antenna could easily be attached to a jacket, vest, belt, or another article of clothing. Furthermore, measurement results of the textile antenna are compared with measurements of a classic, metallic slotted waveguide antenna array showing that the radiation properties of the considered antenna should not be necessarily sacrificed in order to obtain a textile, wearable realization.

The paper is organized as follows. Section 2 presents the design, realization, and experimental characterization of a textile slotted waveguide antenna. The textile prototype is manufactured and compared with the equivalent metallic slotted waveguide antenna, which proved the validity of the presented concept. Section 3 considers the operation of the proposed slotted waveguide antenna on human body phantom. For off-body operation, the antenna design described in Section 2 can readily be used. Next, necessary modifications of the antenna for on-body operation are discussed. Here the goal is to obtain a propagating mode along the body surface. Finally, in-body operation is discussed and necessary modifications for this mode of operation are outlined, since high permittivity and lossy dielectric such as human tissue significantly influence the construction and optimal dimensions of slotted waveguide antenna.

## 2. Experimental Characterization of Textile Slotted Waveguide Antenna

**Figure 1:**
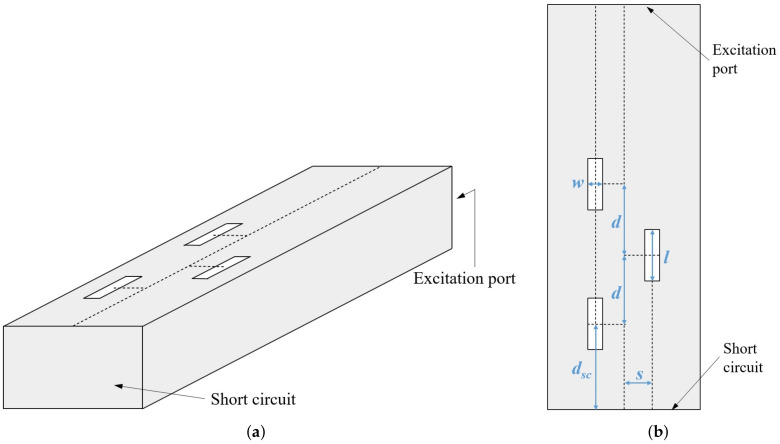
Textile slotted waveguide antenna: (**a**) Side-view, (**b**) top-view.

As a proof-of-concept of the proposed textile slotted waveguide antenna, we have designed and experimentally verified an antenna operating in the 5.8 GHz ISM band. The sketch of the proposed textile antenna is shown in Figure 1. The array contains three slots that are a half guided wavelength apart and approximately half a wavelength long. In order to enlarge the percentage of EM power radiated by each slot, the wavaguide is short-circuited at the distance of three-quarter of the guided wavelength from the center of the third slot. By this, each slot radiates a portion of both a forward and backward propagating wave as if the antenna has six slots instead of physically only three. It should be noted that such a design does not increase the directivity of the antenna, but it does increase the radiated EM power per slot.

The realization of a textile antenna should, of course, include textiles. In our case, the conductive waveguide walls are made of conductive textile and all the connections are sewn together. The radiating slots are cut-out, like in common textile, and their borders are sewn to fix the dimensions and prevent tearing. In other words, we sewed the waveguide together with the slots and the short circuit at the end of the waveguide. Then the sewn waveguide with radiating slots was pulled over an appropriate supporting structure (i.e., over a mould) to keep the desired cross-section of the antenna. Therefore, before making the antenna itself, it was necessary to make three selections: Of a fabric, thread, and of a material for the supporting structure.

For conductive fabric, we selected Shieldex®Nora Dell No.: 1401101S80 [22]. This conductive textile is based on Ni/Cu/Ag-plated polyamide fabric with an average surface resistivity of 0.009 Ω/□ and a thickness of 0.125 mm ±15%. As a conductive thread, the silver-plated metal thread Shieldex®117/17 dtex 2-ply HC + B was chosen [23]. For a mould realization, we considered styrofoam and styrodour. To obtain the rectangular cross-section of the waveguide, the appropriate shape of the mold was obtained by cutting a rectangular prism from the board of considered material. For this reason, styrodour was chosen because it was easier to shape to the required dimensions. However, as the EM properties of the selected styrodour board were not known, it was necessary to determine them experimentally.

**Figure 2:**
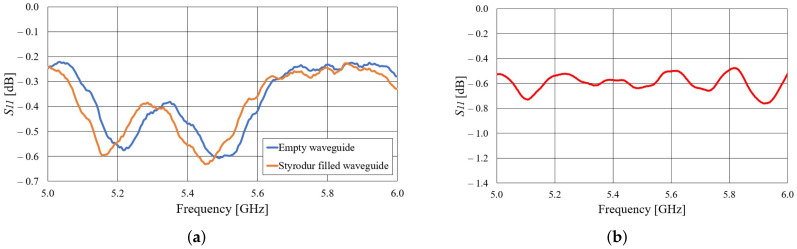
(**a**) Comparison of measured $S_{11}$ parameter of empty and Styrodur-filled short-circuited metallic waveguide. (**b**) Measured $S_{11}$ parameter of short-circuited textile waveguide.

The Styrodur permittivity and losses in the 5.8-GHz ISM band were estimated by inserting a tightly fitting Styrodur filling into a 15-cm long metallic waveguide with a short circuit termination. The comparison of the $S_{11}$ parameter at the waveguide input port for the case with and without Styrodur filing is shown in Figure 2a. From the frequency shift in the position of the minima and maxima of the curves in Figure 2a it is estimated that the permittivity of Styrodur is approximately $\varepsilon_{r}\approx 1.02$. By comparing the measured levels of the two curves in Figure 2a, when the frequency shift is compensated, it follows that the losses introduced by Styroduf filling in comparison to air are negligible. Therefore, Styrodur can be used as a waveguide filling and mould in the waveguide textile antenna.

The next step in the development of a slotted waveguide antenna is to experimentally characterize the textile waveguide itself. For that purpose we have realized a 42-cm long textile waveguide using the considered conductive fabric and styrodour mould. The end of the textile waveguide was short-circuited (like in the proposed antenna design), and the other end was inserted into the WG14 waveguide-to-coax adapter. The measured magnitude of the $S_{11}$ parameter is shown in Figure 2b. It can be seen that the textile waveguide losses are quite small—around 0.75 dB/m in the 5.8 GHz ISM band. The ripples in the $S_{11}$ are due to small a mismatch at the connection of the textile and commercial WG14 waveguide.

The properties of a textile slotted waveguide antenna are demonstrated on a design of a wearable antenna intended for off-body communication mode of operation. The design requirements are as follows:

- The antenna is manufactured from a rectangular waveguide capable of operating in the 5.8-GHz ISM band (thus the WG14 standard was selected);
- Central frequency of operation: 5.85 GHz;
- $S_{11}<-10$ dB in a bandwidth of at least 200 MHz or more;
- Rectangular waveguide slots with the length not exceeding half a wavelength and the width is much smaller than their length (Figure 1).

The dimensions of the slots and distance from the waveguide axis (see Figure 1) were determined using a simple optimization procedure: The $S_{11}$ parameter was forced to obtain the minimum value at the central frequency 5.85 GHz. Such a simple optimization procedure was possible due to presence of short circuit termination, i.e., the desired radiation properties of each slot were combined with an unwanted increase of the reflection coefficient, thus creating optimal balance.

**Figure 3:**
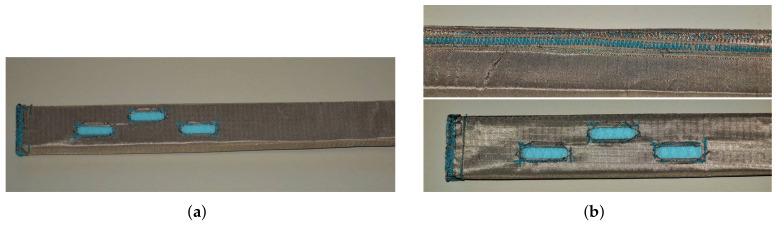
Prototype of the textile antenna (**a**) and details of sewing the waveguide, slots, and short-circuit (**b**).

As a common lock-stitch sewing machine has been used for realization of the textile antenna, the conductive thread was used as the lower thread while the upper thread was a common cotton thread to avoid jamming. Then the slots are cut out and the edges are folded and sewn with the same conductive thread to prevent the fabric from tearing. All other edges of the conductive fabric are also folded and sewn. Finally, the textile antenna is pulled onto a Styrodur mould and inserted into the WG14 waveguide-to-coax adapter. The used flange for connection with the adapter has a bit wider rectangular opening into which the textile waveguide entered tightly, ensuring electric contact between the textile waveguide and metallic adapter. Figure 3 shows the prototype of the textile waveguide antenna together with the details of how it was sewn.

**Figure 4:**
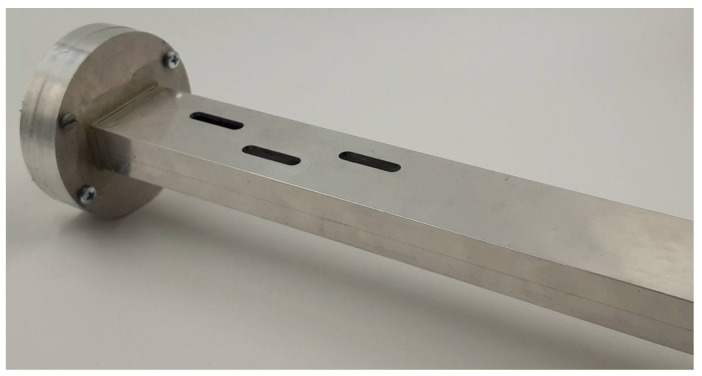
Prototype of the aluminum antenna.

In addition, a reference slotted waveguide antenna made of solid metal was realized in order to fully characterize the proposed textile antenna. For that purpose, we used an aluminum rectangular profile with an inner cross-section 36×16 mm (slightly different than the WG14 standard). The thickness of the aluminum waveguide walls is 2 mm and the slots have round edges to facilitate fabrication. The referent aluminum antenna was manufactured with a computer numerical control (CNC) machine and is shown in Figure 4. Both the textile and referent antenna were modeled with CST Studio Suite and the final antennas dimensions are given in Table 1.

Additionally, Table 2 shows the designed percentage of radiated power per slot.

**Figure 5:**
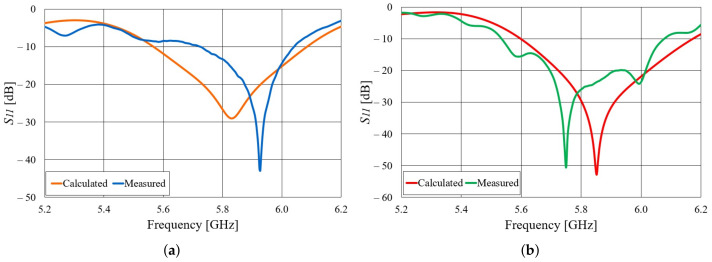
$S_{11}$ parameter of the realized slotted waveguide antennas: (**a**) Textile antenna, (**b**) aluminum antenna.

**Figure 6:**
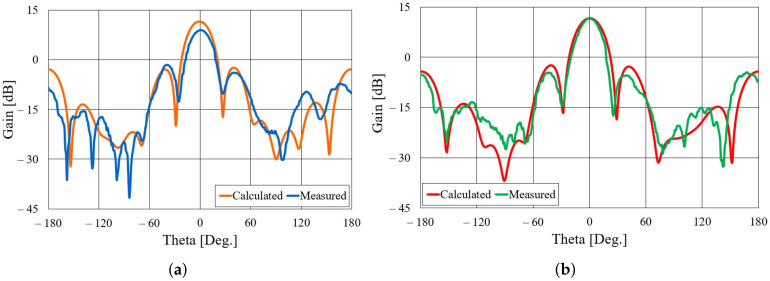
Radiation pattern in the H-plane of the realized slotted waveguide antennas: (**a**) Textile antenna, (**b**) aluminum antenna.

**Figure 7:**
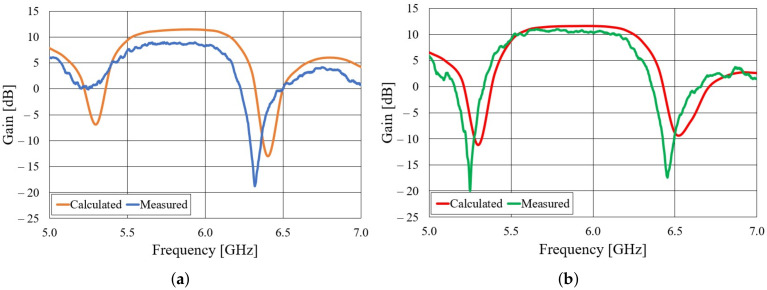
Realized gain of the slotted waveguide antennas: (**a**) Textile antenna, (**b**) aluminum antenna.

Both realized antennas are experimentally characterized in an anechoic chamber. The $S_{11}$ parameters of the aluminum and textile antennas, compared to the $S_{11}$ simulated in CST Studio Suite, is shown in Figure 5. Both the aluminum and textile antenna are matched in a frequency band of more than 300 MHz. In the case of the aluminum antenna, there are ripples in the measured $S_{11}$ curve, probably due to the difference in the cross-section of the adapter (WG14 standard) and the waveguide with slots. One can also observe a frequency shift in the minimum of the $S_{11}$ curve. In the case of the textile antenna, this shift is due to a limited accuracy in the realization of the designed dimensions of the slotted waveguide array (this accuracy can be increased with professional machine fabrication). By subsequent measurements, the accuracy of 0.5 mm in the realization of the antenna array was determined. The most critical dimensions for the frequency response are the distance between the slots and width of the waveguide (the realized dimensions are shorter by 0.5 mm). With realized antenna dimensions, the minimum in the calculated $S_{11}$ curve is shifted to 5.93 GHz. The radiation patterns of both antennas in the H-plane at 5.85 GHz are shown in Figure 6. The agreement between calculated and measured radiation patterns for both aluminum and textile antenna is very good. The realized gain in the direction of maximum radiation is shown in Figure 7. It can be seen that the realized gain is stable for both antennas within a wide operating band, with the textile antenna providing only around 2 dB less gain. In other words, the radiation efficiency of the textile antenna is between 50% and 63% within the frequency band of interest. The lower radiation efficiency is mostly due to imperfections of the textile realization of the slotted waveguide array (losses in the fabric, roughness of the surface in particular around slots, and leakage of energy through the fabric).

## 3. Antennas for Body-Centric Wireless Communications

Body-centric wireless communication systems have many potential applications, thus becoming the important part of novel communication systems (e.g., under the 5G scheme). We can distinguish three basic types of communication channels: Off-body, on-body, and in-body [5]. In this section, we will discuss design requirements on textile slotted waveguide antennas if we would like to use them for these three electromagnetically different scenarios.

### 3.1. Off-Body Communication

**Figure 8:**
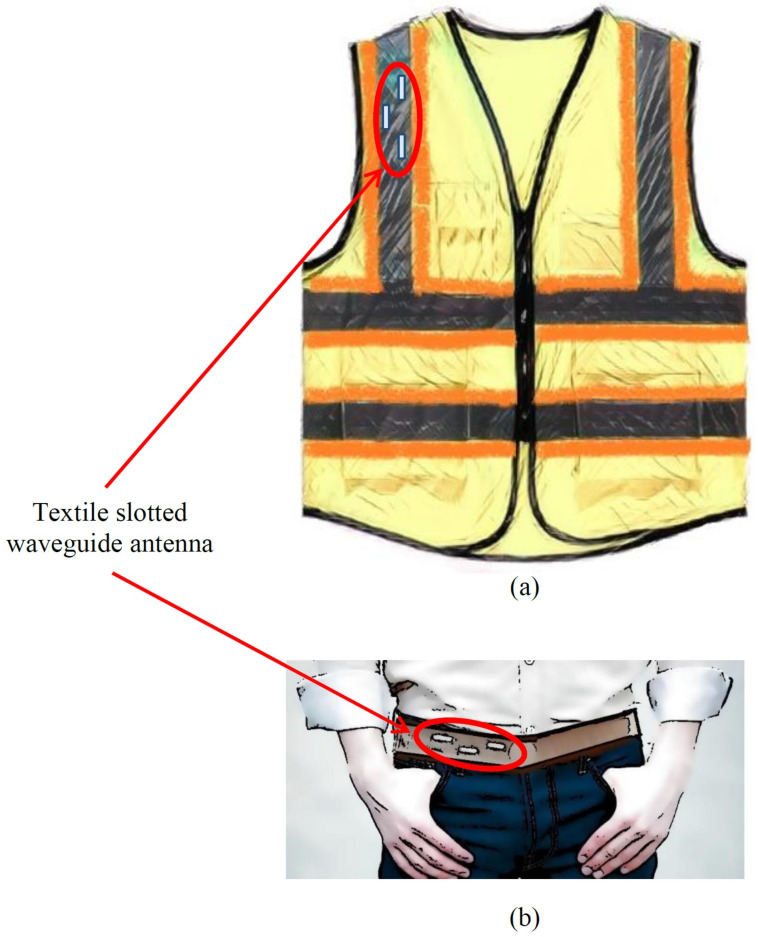
The textile antenna in use: (**a**) Smart safety vest, (**b**) smart belt.

Figure 8 illustrates a possible end product of the waveguide textile antenna. It is evident that the antenna can be part of the uniform (e.g., for firemen and other people involved in everyday rescue operations) or smart vest (for people working in different working environments—just to mention production lines or warehouses in which an off-body communication system enable robots and humans to (co)operate safely and flexibly [24]). Therefore, the basic requirement is to establish a communication link with the access point located somewhere in mostly an indoor working environment.

**Figure 9:**
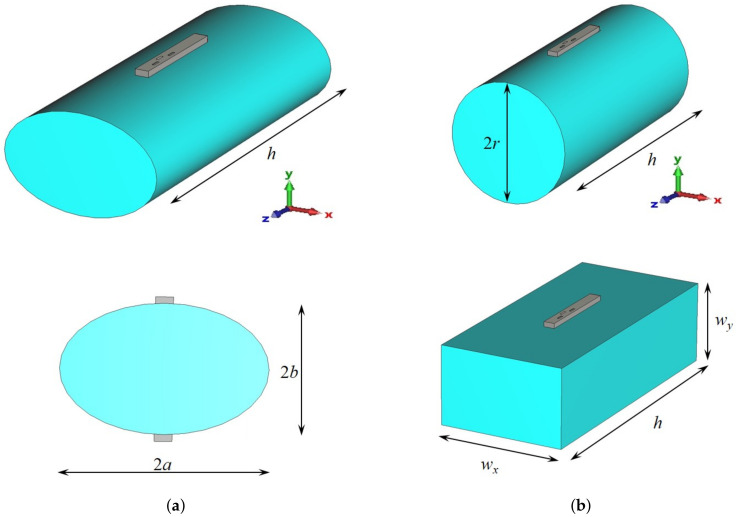
Sketch of the cylindrical human Phantom with waveguide antenna: (**a**) phantom with an elliptical cross-section (a=200 mm, b=125 mm, h=837 mm), (**b**) phantoms with a circular and rectangular cross-section (r=200 mm, h=837 mm, $w_{x}=200$ mm, $w_{y}=125$ mm).

**Figure 10:**
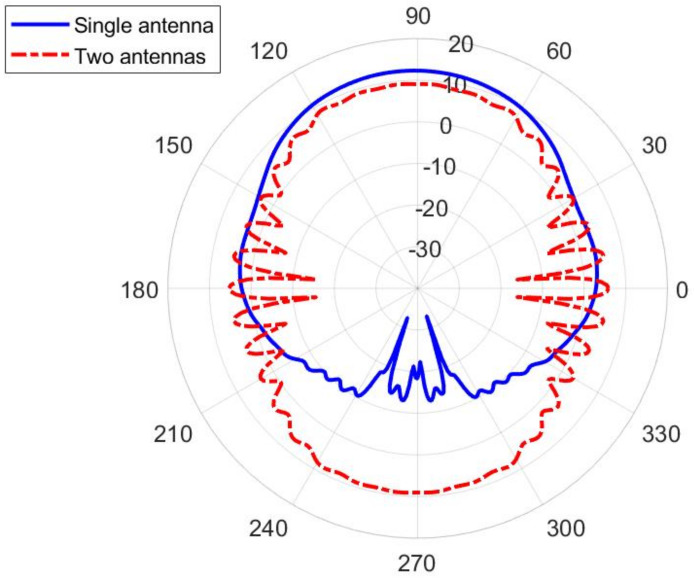
Radiation pattern in the E-plane (xy-plane) of the textile slotted waveguide array placed vertically at the human phantom of the elliptical cross-section (Figure 9a). Both the cases of one and two slotted waveguide arrays (having excitation in phase) are shown. Radial scale is in dB, angular is in degrees.

In order to establish connection with an arbitrary positioned access point, an omnidirectional type of radiation pattern is required in the horizontal plane with a moderate beamwidth in the vertical plane. Therefore, we can discuss two possible topologies of using textile waveguide antennas—as a part of a smart vest (in which the waveguide is vertically oriented), and as a part of a smart belt (where the waveguide is horizontally oriented). In order to discuss the presence of the human body, we selected a homogeneous cylindrical model of a human body having permittivity and conductivity given by the IEEE human body model at a working ISM band ($\varepsilon_{r}=48.2$ and σ=6 S/m, [12]). The phantom has an elliptical cross-section with the semi-major and semi-minor axes equal to 200 mm and 125 mm, respectively (Figure 9). Figure 10 shows the radiation pattern of a waveguide antenna designed in Section 2 and placed vertically on a human phantom. Two different scenarios are considered: Having one antenna only (on the front or back side of the smart vest), or having two antennas (both on the front and back side). It is evident that for establishing the link with a high availability requirement two antennas are needed. However, to be sure to avoid nulls mostly in a lateral direction, it is preferable to use a frequency type of multiplexing, i.e., waveguide antenna arrays working on different frequencies (i.e., using different communication channels).

**Figure 11:**
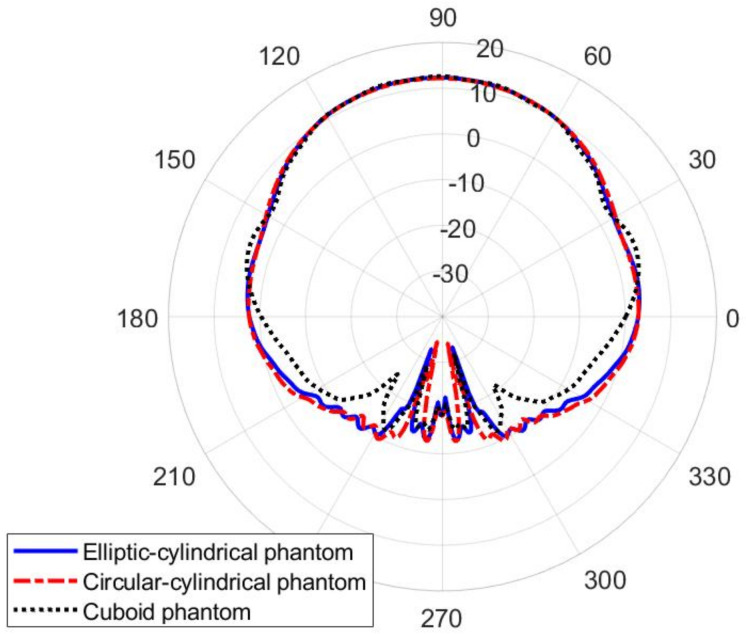
Influence of the human phantom cross-section on the radiation pattern in the E-plane (xy-plane) of the textile slotted waveguide array. Radial scale is in dB, angular is in degrees.

The influence of the shape of the human phantom is discussed in Figure 11. The considered phantomes have an elliptical, circular, and rectangular cross-section, as illustrated in Figure 9. It can be seen that the radiation pattern of the proposed textile waveguide antenna is not sensitive on the body shape, thus demonstrating the robustness of the proposed design.

To evaluate the exposure of the antenna user to the electromagnetic fields, the SAR values have been calculated and listed in Table 3 for the case of a circular-cylindrical phantom. The obtained values are very small, thus the proposed antenna is a good candidate for off-body communication systems also from the maximum exposure limit point of view.

**Figure 12:**
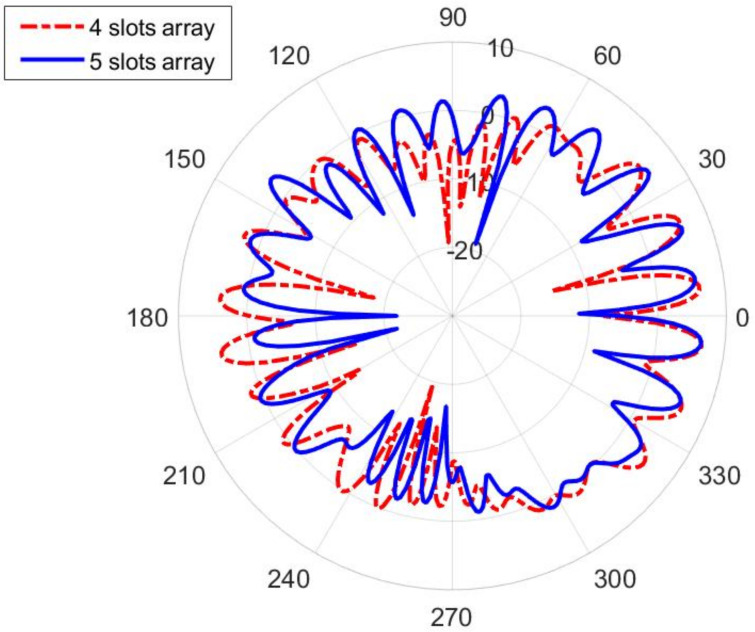
Radiation pattern in the H-plane (xy-plane) of the slotted waveguide array placed horizontally on the human phantom with a circular cross-section (smart belt application). Both cases of the array with four slots (positioned at coordinates $\varphi =30^{\circ },150^{\circ },210^{\circ }$, and $330^{\circ }$) and with five slots (positioned at coordinates $\varphi =30^{\circ },150^{\circ },180^{\circ },210^{\circ }$, and $330^{\circ }$) are shown. Radial scale is in dB, angular is in degrees.

**Figure 13:**
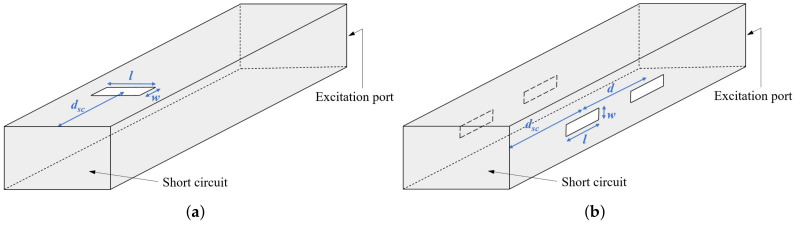
Alternative slotted waveguide antenna designs: (**a**) Waveguide antenna with one transverse slot, (**b**) waveguide array with two pairs of longitudinal slots at both narrow waveguide walls.

**Figure 14:**
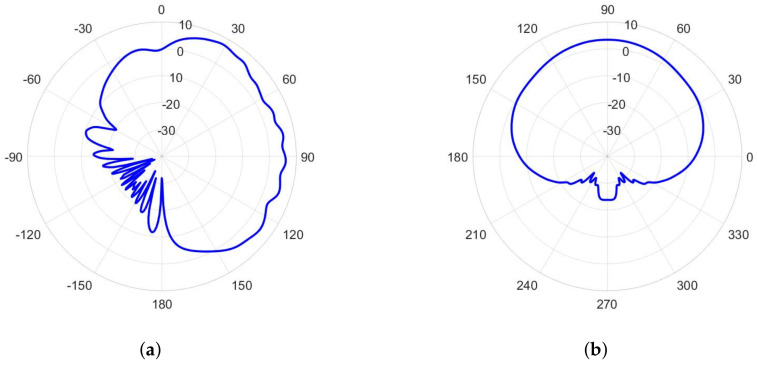
Radiation pattern of the waveguide with single transverse slot placed horizontally on the human phantom with a circular cross-section (smart belt application, Figure 13): (**a**) yz-plane pattern (H-plane), (**b**) xy-plane pattern (E-plane). Radial scale is in dB, angular is in degrees.

The smart belt type of application (in which the slots are horizontally positioned) suggest two approaches to the design—using a Distributed Antenna System (DAS) approach [25], where the slots are distributed along the smart belt, or using single slot antennas (it is enough to have two of them, each radiating on each side of the body). In the first case, since the distance of the slots is not large enough (like it is assumed in the DAS approach), the radiation pattern has many nulls, see Figure 12. However, single slot design offers much better coverage. In order to establish an efficient antenna, we have designed a transverse slot antenna, together with the short circuit at the distance of three-quarter of the guided wavelength, see Figure 13a. The optimized dimensions are: l=26 mm, w=11.8 mm, and $d_{sc}=41.4$ mm (as before, the optimization is based on minimizing the magnitude of $S_{11}$ parameter at central frequency 5.85 GHz). The radiation pattern of such an antenna, in the presence of the circular-cylindrical phantom, is shown in Figure 14.

### 3.2. On-Body Communication

**Figure 15:**
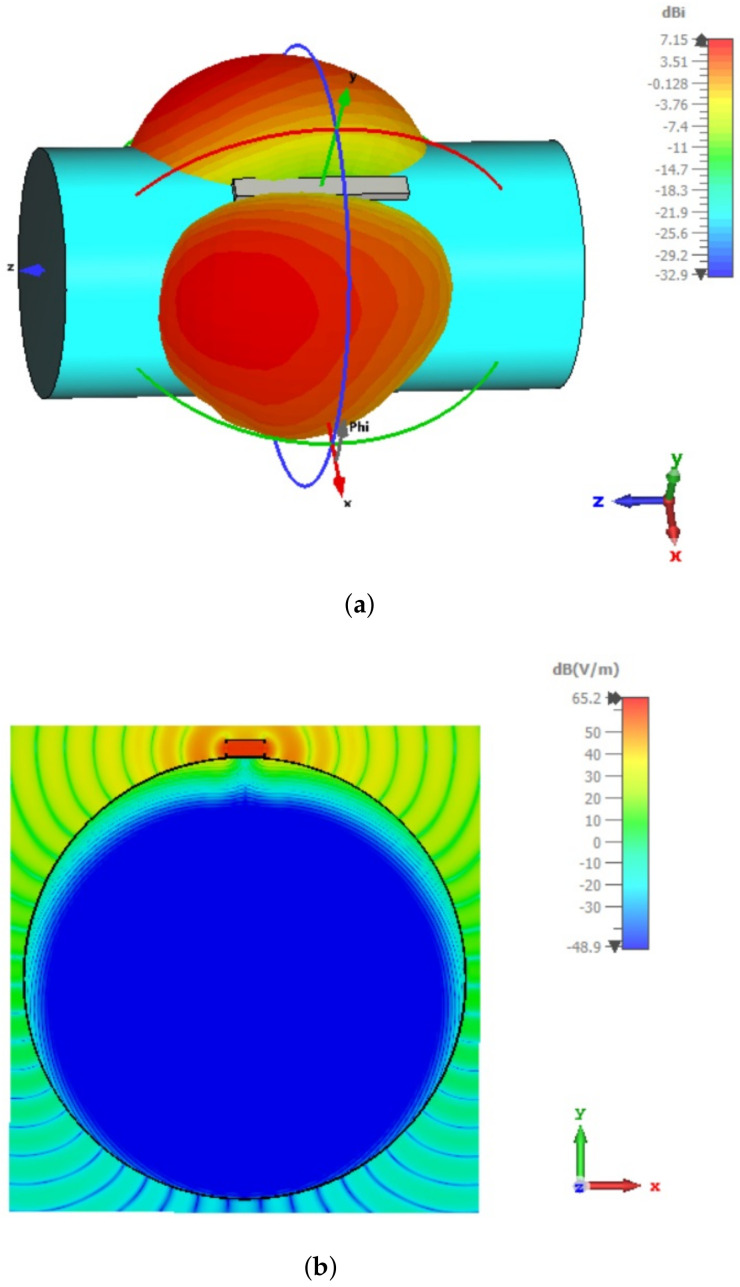
3D radiation pattern (**a**) and moment-in-time snapshot of the E-field distribution in the plane perpendicular to the waveguide slots (**b**) of the waveguide array with one pair of slots shown in Figure 13b. The maximum of the E-field in the human phantom, and thus the maximum of SAR, is located next to the waveguide edge where the slots are located.

In several studies, it was shown that a much larger coupling between two on-body antennas is obtained if the EM wave, propagating along the body surface, is normally polarized, i.e., if the direction of the E-field is normal to the body surface (see e.g., [26,27,28]). Therefore, we proposed the modified design of textile slotted waveguide antenna, as shown in Figure 13b. The slots are now cut in the narrow waveguide wall resulting in launching the normally polarized EM wave along the body. The optimized dimensions of the waveguide array containing one pair of longitudinal slots are: l=49.7 mm, w=12 mm, and $d_{sc}=56.8$ mm. The example of the surface radiation pattern of such an array is shown in Figure 15 where both the 3D radiation pattern and the moment-in-time snapshot of the E-field distribution in the plane perpendicular to the phantom are presented (the input power at waveguide port is 0.5 W). The Figure reveals that for a normally polarized excitation (i.e., due to slots in the narrow waveguide walls) the EM wave travels along the surface of the body which enables on-body communication. Additionally, the penetration depth of EM fields into the human phantom is very small. The SAR values for this case are larger comparing to the off-body antenna design (see Table 3); however the increase is not so large. As the maximum of SAR is located near the edge of the waveguide (see Figure 15b), the SAR values can be reduced by sewing a strip of conductive fabric next to the edge of the waveguide. This conductive strip will not “spoil” the propagation of the EM wave around the body since it supports propagation of the normally polarized EM wave.

### 3.3. In-Body Communication

**Figure 16:**
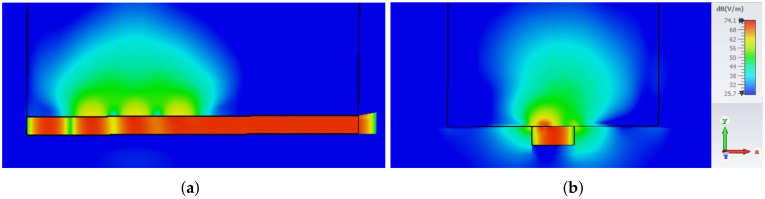
Antenna radiating into the human body—distribution of the E-field magnitude: (**a**) H-plane cross-section, (**b**) E-plane cross-section. The black frame indicates the cuboid phantom.

**Figure 17:**
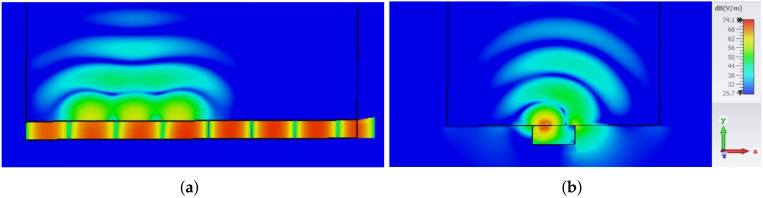
Antenna radiating into the human body—snapshot of a moment in time of the E-field distribution: (**a**) H-plane cross-section, (**b**) E-plane cross-section. The black frame indicates the cuboid phantom.

For in-body applications, it is necessary to take into account the fact that the antenna is radiating into a human body, i.e., into the lossy media with large values of permittivity and conductivity. Therefore, the modified slotted waveguide array design is based on filling the waveguide with material with similar permittivity to the one of the human body, i.e., with semisolids such as agar and silicone rubber or with solids such as ceramics [29]. If we use the filling with permittivity $\varepsilon_{r}=48.2$, then the modified dimensions of the waveguide array are 5.02×2.28 mm${}^{2}$ (obtained by the optimization process). Note that these dimensions are in principal scaled ones of the antenna in Figure 1 and Table 1 by factor $\sqrt{\varepsilon_{r}}$. The designed antenna is matched at 5.85 GHz, and the optimized dimensions of the waveguide array with three slots are: l=3.92 mm, w=0.69 mm, d=5.47 mm, s=1.03 mm, and $d_{sc}=8.21$ mm. Penetration of the EM energy into the human body is illustrated in Figure 16 and Figure 17. In order to visualize the EM wave propagation inside and in the vicinity of the human phantom, both the magnitude and the moment-in-time snapshot of the E-field distribution are given (the input power at waveguide port is 0.5 W). Figure 16 and Figure 17 also reveal that the amplitude of the electric field inside the phantom decays rapidly due to the losses in the tissue, e.g., at a depth of 50 mm, the electric field decreases by more than 20 dB in comparison to the value at the waveguide slot (in the direction along the body surface, the decrease of the E-field amplitude is much larger). In addition, some portion of the energy is radiated from the surface of the body since for such lossy body there is no critical angle related to the ray picture of propagation in the human phantom (like it would be in the case of lossless body), see e.g., [30].

The calculated SAR values follows the E-field distribution inside the human phantom. The obtained SAR values are much larger in comparison to the off-body and on-body designs (Table 3), thus proving that the proposed antenna design is an efficient launcher of EM waves into the human body and can be used for medical applications, such as microwave radiotherapy.

## 4. Conclusions

In this paper a new type of textile antenna, slotted waveguide array, is proposed and experimentally characterized in the 5.8 GHz ISM band. The measured operating bandwidth is larger than 300 MHz. As a reference case, the equivalent aluminum-slotted waveguide antenna is fabricated, showing similar measured parameters, except with a difference in the realized gain of approximately 2 dB. Nevertheless, the radiation efficiency of the textile antenna is larger than 50% in the whole operating bandwidth. The reduced gain of is mostly due to imperfections of the textile—roughness of the surface and energy leakage through the fabric. The sewing procedure required in the antenna manufacturing process is also elaborated in detail, providing the complete proof-of-concept for the developed textile antenna. The proposed textile antenna on account of its lightweight but robust characteristics can be integrated into clothing to create e.g., a smart uniform, belt, or safety vest. Furthermore, different realizations of the slotted antenna are also presented—longitudinal or transverse slots placed on a broad or narrow waveguide wall—which makes the proposed antenna suitable for all three (off-body, on-body, and in-body) body-centric communication scenarios.

## Figures and Tables

**Table 1:** The antenna dimensions.

	Textile	Aluminium
Slot length *l*	24.5 mm	26.4 mm
Slot width *w*	6.0 mm	6.0 mm
Slot offset *s*	5.5 mm	6.12 mm
Slot spacing *d*	38.0 mm	36.5 mm
Distance from SC $d_{sc}$	57.0 mm	54.8 mm

**Table 2:** Percentage of radiated power per slot.

	First Slot	Second Slot	Third Slot
Incident radiated power	24.82%	18.66%	14.03%
Reflected radiated power	5.96%	7.93%	10.55%
Total radiated power	30.78%	26.59%	24.57%

**Table 3:** Simulated maximum SAR value for 1 W antenna input power averaged per 1 g and 10 g of tissue, and corresponding input power $P_{in}$ for which the exposure limit of 2 W/kg is reached [13,14].

	1 g SAR (W/kg)	10 g SAR (W/kg)	$P_{\mathrm{in}}$ (mW)
Off-body design	1.12	0.46	4348
On-body design	17.76	5.59	358
In-body design	583.64	92.36	22
